# The Performance of Partial Least Squares Methods in Virtual Nanosensor Array—Multiple Metal Ions Sensing Based on Multispectral Fluorescence of Quantum Dots

**DOI:** 10.3390/ma17194766

**Published:** 2024-09-28

**Authors:** Klaudia Głowacz, Mikołaj Cieślak, Patrycja Ciosek-Skibińska

**Affiliations:** Chair of Medical Biotechnology, Faculty of Chemistry, Warsaw University of Technology, Noakowskiego 3, 00-664 Warsaw, Poland; klaudia.glowacz@pw.edu.pl (K.G.);

**Keywords:** quantum dots, metal ions, excitation–emission matrix fluorescence spectroscopy, multispectral fluorescence, PLS analysis, multi-analyte detection, multi-responsive probe, multiple-response receptor, multi-parameter sensor, virtual sensor array

## Abstract

The design of chemical sensors and probes is usually based on selective receptors for individual analytes, however, many analytical tasks are dedicated to multi-analyte sensing or recognizing properties of the sample related to more than one analyte. While it is possible to simultaneously use multiple sensors/receptors in such cases, multi-responsive probes could be an attractive alternative. In this work, we use thiomalic acid-capped CdTe quantum dots as a multiple-response receptor for the detection and quantification of six heavy metal cations: Ag(I), Cd(II), Co(II), Cu(II), Ni(II), and Pb(II) at micromolar concentration levels. Multiplexing is realized via multispectral fluorescence (so-called virtual sensor array). For such a sensing strategy, the effective decoding of the excitation–emission spectrum is essential. Herein, we show how various parameters of chemometric analysis by the Partial Least Squares method, such as preprocessing type and data structure, influence the performance of discrimination and quantification of the heavy metals. The established models are characterized by respective performance metrics (accuracy, sensitivity, precision, specificity/RMSE, a, b, R^2^) determined for both train and test sets in replicates, to obtain reliable and repeatable results.

## 1. Introduction

Quantum dots (QDs) are semiconductor nanocrystals, which, due to their unique photoluminescent properties, have become attractive nanomaterials used in biomedical applications, including bio-labeling, bio-imaging, and bio-targeting [1,2,3]. Moreover, as a consequence of their versatile surface chemistry and ligand binding ability, QDs have also been exploited in analytical chemistry, primarily as optical sensors for the detection of small molecules. Since metal ions are an essential analytical target in, e.g., environmental or biological samples [4,5,6], it is not surprising that many QD-based optical sensors have been developed over the years for their detection [2,7,8,9,10,11].The use of quantum dots for the qualitative/quantitative analysis of metal ions is most often based on the observation of the change in their fluorescence signal under the influence of selective interaction with an analyte [2]. However, applying such an approach to detecting multiple analytes in a sample requires the design of a highly selective receptor element for each analyte, thus representing a major synthetic challenge. To resolve this issue, an alternative sensing strategy might be employed, namely pattern-based sensing, which relies on the design of receptors that differentially interact with various analyte components [12,13,14,15] or, in some cases, on the application of one sensor in combination with detection techniques that provides multidimensional information about the composition of the tested sample [16,17]. The analytical signals obtained in this way form chemical “fingerprints” that consist of many variables and often contain complex information about several components of the sample at the same time [14,18]. Therefore, their use in chemical analysis must be carried out using advanced numerical tools, i.e., chemometric/machine learning methods, which allow the extraction of analytically useful information for qualitative or quantitative analysis [19,20]. Chemometric analysis is a multi-stage and iterative process, the course of which may vary depending on prior knowledge about the system under study, the type and structure of chemical data, as well as the research goals set. Therefore, a thorough optimization of numerical data processing is an inherent and essential element of the design of pattern-based sensing methods. An insightful description of the chemometric models for data processing in quantum dots-based sensing systems can be found elsewhere [21].

The photoluminescent properties of quantum dots are modulated by various chemical and physical processes occurring on the nanocrystal’s surface when metal ions are in its vicinity. These processes affect QDs’ surface charges and trap states, which might lead to different quenching mechanisms of the nanocrystal. As a result, diverse changes can be observed in a fluorescence spectrum of QDs in the presence of metal ions [2,22]. Due to the complex mechanisms of QD-analyte interactions, these nanocrystals can be regarded as multi-analyte receptors. They can be applied like specially designed multi-responsive fluorescence probes dedicated to the simultaneous detection of multiple analytes, i.e., multiplexing [10,11,23,24]. Such an approach greatly simplifies the multi-analyte sensing system providing a virtual sensor/receptor array. Moreover, it allows problems linked with different uptake, localization, and metabolism profiles to be avoided when the traditional single-functional probes for various analytes are used in cellular biology research [23]. However, the development and synthesis of such probes are extremely challenging because multiple recognition sites and reporting groups should be incorporated into a single molecule [25]. Until now, various strategies for multiplexed determination of heavy metal ions based on QDs probes have been proposed [13,14,21,26]. They were based on fluorometric, colorimetric, or electrochemical transduction applied to QDs with various cores (chalcogenide QDs, carbon QDs, graphene QDs, etc.), and various ligands (mercaptopropionic acid, glutathione, thioglycolic acid, etc.) [26]. Although thiomalic acid-capped CdTe QDs were used for the synchronous detection of Hg^2+^ and Cu^2+^, the method was based on diverse fluorescence quenching and the recovery constant between Hg^2+^ and Cu^2+^, without considering chemometric approaches [27].

In our case, to realize multiple-metal ion recognition using only a single type of quantum dots, we decided to apply a multispectral mode of fluorescence measurements, i.e., excitation–emission matrix (EEM) fluorescence spectroscopy, allowing us to capture multidimensional information on these metal ion-induced changes in photoluminescence. As a consequence of such a sensing strategy, multi-analyte sensing through only one probe type can be attempted. As we have shown in our previous work [17], the EEM fluorescence response of QDs provides a significant improvement of analyte discrimination in comparison to zeroth-order (scalar–fluorescence intensity/shift) or first-order (vector–spectrum) fluorescence response. This effect was explored on the exemplary qualitative and quantitative analysis of catecholamines interacting with glutathione-capped CdSeS/ZnS quantum dots [17]. It must be emphasized that possibly even better sensing results could be achieved by optimizing the preprocessing step of data analysis or with the application of procedures dedicated to second-order data structure, such as n-way Partial Least Squares (N- PLS) [28].

**Figure 1:**
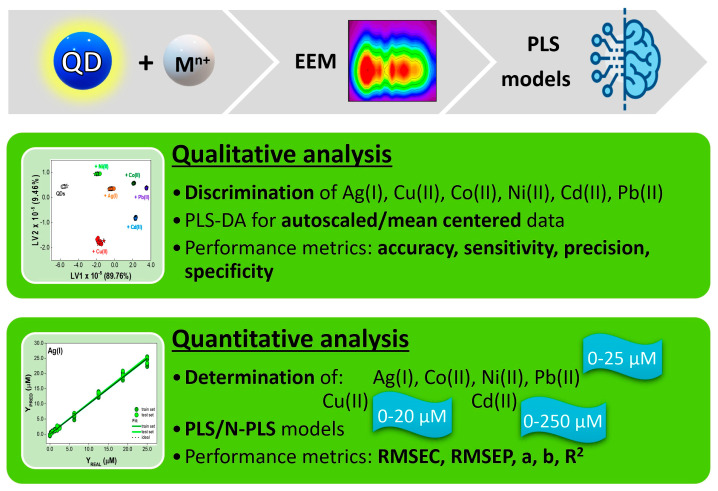
Scheme of the workflow of qualitative and quantitative analysis of six metal ions by multi-responsive QDs with multispectral fluorescence detection.

Therefore, in the framework of this study, we applied various variants of Partial Least Squares (PLS) methods for the pattern-based sensing of metal ions employing thiomalic acid-capped CdTe quantum dots. We investigated how data structure (unfolded vs. multiway) and/or preprocessing steps influence the performance of the qualitative and quantitative analysis of six metal ions sensed by a single probe but with multispectral detection, enabling multi-analyte sensing, and thus virtual receptor array. The workflow is presented in Figure 1.

## 2. Materials and Methods

### 2.1. Reagents and Materials

Commercially available thiomalic acid (TMA)-capped quantum dots (QDs) with a CdTe core, a diameter of 3.06 nm, and λ_max_ = 560 nm obtained from PlasmaChem GmbH (Berlin, Germany) were used as fluorescent, multi-responsive receptors for metal ion detection and determination in this work. Nitrate salts of Ag(I), Cd(II), Co(II), Cu(II), Ni(II), and Pb(II), as well as N′-(2-ethane sulfonic acid) (HEPES), were obtained from Sigma-Merck (Poznań, Poland). Milli-Q water was used for the preparation of all aqueous solutions. All reagents were used as received.

### 2.2. Qualitative Analysis Experiments

All stock solutions used in the qualitative analysis experiments, including QDs and solutions of Ag(I), Cd(II), Co(II), Cu(II), Ni(II), and Pb(II) nitrate salts, were prepared in 50 mM HEPES buffer at pH 7.4. Samples for EEM measurements containing metal ions were prepared in 96-well plates by adding 50 µL of appropriate metal ion solution into 150 µL of QDs solution. The concentration of QDs in a well was 20 µg/mL, while the concentration of metal ions was 20 µM for Cd(II), Co(II), Ni(II), Pb(II), or 1 µM for Ag(I) and Cu(II), respectively. Control samples were prepared as solutions containing only QDs, i.e., instead of metal ion solution, 50 µL of 50 mM HEPPES buffer at pH 7.4 was added. The final volume of every sample was 200 µL. The samples containing metal ions were prepared and measured in 11 replicates, while QDs control samples were measured in 8 replicates.

### 2.3. Quantitative Analysis Experiments

Since at higher pH values, the precipitation of metal hydroxides is possible, to expand the concentration range for the quantitative analysis of metal ions, a lower pH of 6.5 was chosen. Therefore, before quantitative analysis experiments, stock solutions of Ag(I), Cd(II), Co(II), Cu(II), Ni(II), and Pb(II) nitrate salts, as well as QDs, were prepared in 50 mM HEPES buffer at pH 6.5. Separate 96-well plates were prepared for each metal ion, where samples at 10 concentration levels were included. Similarly to quantitative analysis experiments, samples were prepared by adding 50 µL of metal ion solution and 150 µL of QDs solution. The concentration of quantum dots was selected in such a way that the recorded signal of control samples (only containing QDs) in quantitative analysis experiments was the same as in case of qualitative analysis, and was 45 µg/mL. The concentration range of metal ions depended on the type and the degree of induced changes in the EEM spectrum of quantum dots, and was 0–25 µM for Ag(I) and Cu(II), 0–250 µM for Co(II), Ni(II), and Pb(II), and 0–2500 µM for Cd(II). Each sample type was prepared and measured in 8 replicates.

### 2.4. EEM Fluorescence Measurements

All samples for EEM fluorescence measurements were prepared in UV-Star 96-well plates (Greiner Bio-One GmbH, Kremsmünster, Austria), and fluorescence measurements were performed with Synergy Neo 2 Hybrid Multi-Mode Microplate Reader fluorescence spectrometer (BioTek Instruments, Inc., Winooski, VT, USA). Before the acquisition of EEM data, the samples were mixed for 1 min. Then, EEMs were collected using a hand-written protocol consisting of recording consecutive emission spectra at decreasing excitation wavelengths. The spectral range of excitation was 250–500 nm (resolution of 10 nm), while the emission range was 520–700 nm (resolution of 1 nm). All experiments were performed at room temperature.

### 2.5. Data Pretreatment and Chemometric Analysis

The performed fluorescence measurements resulted in a collection of 26 emission spectra (data vectors) per sample, which were further arranged in excitation–emission matrices with a hand-written protocol in the Python programming language. Each sample was characterized by EEM of dimensions 181 × 26 (λ_em_ × λ_ex_). Unfolded Partial Least Squares-Discriminant Analysis (PLS-DA) was used for the qualitative analysis of metal ions, and its performance with two preprocessing methods was compared, namely autoscaling and mean centering. Before PLS-DA modeling (Scheme S1 in ESI), EEM describing each sample was unfolded into a data vector by combining the excitation and emission mode (1 × [λ_ex_ × λ_em_]) by the hand-written protocol in the Python programming language. The dimension of the resulting data vector for each sample was 1 × 4706, and the data matrix subjected to PLS-DA analysis was of 74 × 4706 (samples × [λ_ex_ × λ_em_]). For the quantitative analysis of metal ions, the performance of unfolded Partial Least Squares (PLS) was compared with n-way Partial Least Squares (N-PLS, Scheme S2 in ESI). The data applied to the unfolded PLS model development were prepared in the same manner as in the case of unfolded PLS-DA, resulting in a data matrix of dimension 80 × 4706 for each metal ion (10 concentration levels × 8 replicates × [λ_ex_ × λ_em_]). Before PLS model development, unfolded EEMs were preprocessed by mean centering. For metal ions inducing the changes in the baseline, the performance of PLS models established with autoscaled data was also compared. N-PLS was performed on the multiway arrays of dimensions 80 × 181 × 26 (samples × λ_em_ × λ_ex_). In this case, EEM spectra were normalized to the emission maximum of QDs as a data pretreatment step. For both qualitative and quantitative analysis, prepared data matrices were randomly split into train and test data sets in 3 replications independently to assess the repeatability of each model (65% of the samples were used for model calibration and 35% for validation). Thus, the mean value and standard deviation of the results obtained using 3 different train and test sets were presented. To select the correct number of components describing each model, a cross-validation of venetian blinds was used.

### 2.6. Software

All preprocessing and chemometric analyses were performed in commercially available Solo 9.1 (Eigenvector Research Inc., Manson, WA, USA) software and supported by hand-written protocols in Python 3.x. Figures were generated using Origin Pro 2021b (OriginLab Corporation, Northhampton, MA, USA) software.

## 3. Results

### 3.1. Qualitative Analysis of Metal Ions

**Figure 2:**
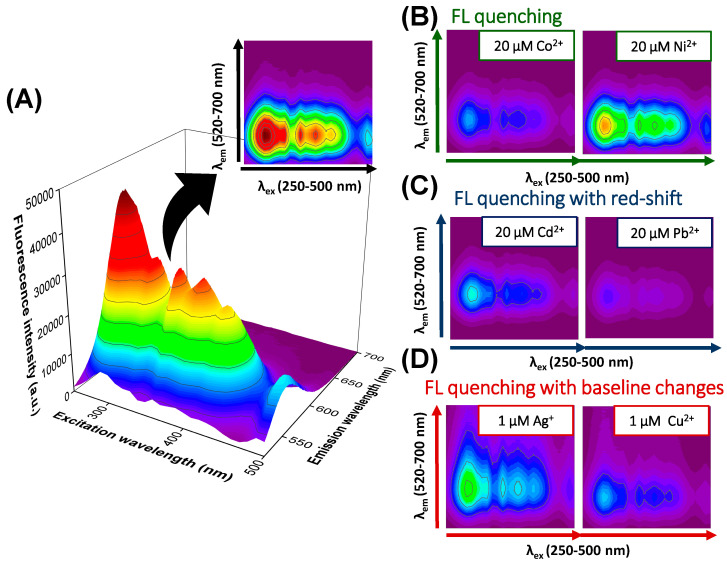
The influence of metal ions on the EEM fluorescence spectrum of QDs (20 µg/mL, 50 mM HEPES buffer, pH 7.4). (**A**) EEM spectrum of QDs. (**B**–**D**) EEM spectra of QDs in the presence of metal ions showing their diverse impact on QD’ fluorescence (FL) signal.

Due to the fundamental role of metal ions in various biological processes and environmental studies, they have become essential analytes for many analytical platforms, including optical QD-based sensors. Interestingly, depending on the structure of the QD’s core, surface-capping ligands, or metal ion, and consequently the mechanism of QDs–metal ion interaction, differential changes in the fluorescence response can be expected [2]. Indeed, under the selected measurement conditions, the tested metal ions induce three effects visible in the excitation–emission matrix of QDs, which is shown in Figure 2. Co(II) and Ni(II) ions cause only a decrease in the fluorescence intensity of nanocrystal over the entire spectral range (Figure 2A). The degree of fluorescence quenching differs for both ions, with a more pronounced effect in the case of Co(II). In the presence of Cd(II) and Pb(II) ions, the fluorescence quenching of QDs is also accompanied by the red-shift of the emission maxima (Figure 2B). One of the possible causes of fluorescence quenching by metal ions is their binding to the QDs surface ligand and the change of surface properties such as surface charge. The second possibility is the formation of QD agglomerates due to the high affinity of metal ions and surface ligands. The displacement of the ligand under the influence of the metal may cause a gap in the surface of the QDs and, as a result, the formation of nanoparticle agglomerates. In the absence of spectral shifts in the fluorescence emission maximum, static quenching can be expected (which may be caused by the electrostatic interaction of the ligand and the metal ion), while when signal shifts are observed, dynamic quenching is possible (e.g., caused by the agglomeration phenomenon) [2]. The most complex influence on the EEM fluorescent response of QDs can be noted for Ag(I) and Cu(II) ions, where, in addition to fluorescence quenching and a slight red-shift, changes in the baseline in the emission range of 600–650 nm can be observed (Figure 2C). These observations might be associated with the interaction of metal ions with the QD surface, leading to changes in the charge transfer mechanism [2]. As part of this work, we decided to check whether the observed effects are sufficient for the qualitative analysis of these analytes performed by processing the entire excitation–emission matrices with chemometric tools.

For the qualitative analysis of metal ions, unfolded Partial Least Squares-Discriminant Analysis (PLS-DA) was selected as a versatile chemometric method, widely used for spectral data processing. This linear classification method relies on the determination of latent variables (LVs), which are linear combinations of original variables describing a maximum covariance between X data matrix, e.g., spectral information describing tested samples (descriptive variables) and Y target matrix, i.e., the class membership of these samples (qualitative variables). The undoubted advantage of the PLS-DA method in the context of spectral data processing is that it allows the interpretation and understanding of relations between descriptive and qualitative variables by analyzing LV’ scores (representing the co-ordinates of samples in the LV projection hyperspace) and loadings (showing the impact of original variables on the corresponding latent variable) [29]. This algorithm is straightforward, linear, and robust, especially useful for analyzing strongly collinear and noisy data, even with highly multidimensional input. Due to the different types of influence of investigated metal ions on the EEM spectra of QDs, the comparison of two preprocessing methods for the extraction of discriminatory information was realized, namely mean centering (the standard method of spectral data processing) and autoscaling [30].

The number of latent variables in each model was established based on the minimalization of the classification error of cross-validation. The decision on class affinity of samples representing train and test sets was made according to the ‘most probable’ rule, which means that the sample was assigned to the class for which the highest probability was obtained in the output vector. The performance of PLS-DA models established with different data pre-treatment steps was evaluated based on accuracy, sensitivity, precision, and specificity (see Table S1 in Electronic Supplementary Information (ESI)). These quality performance metrics were calculated based on confusion matrices providing numbers of true negatives (TN), false negatives (FN), false positives (FP), and true positives (TP) [31].

**Figure 3:**
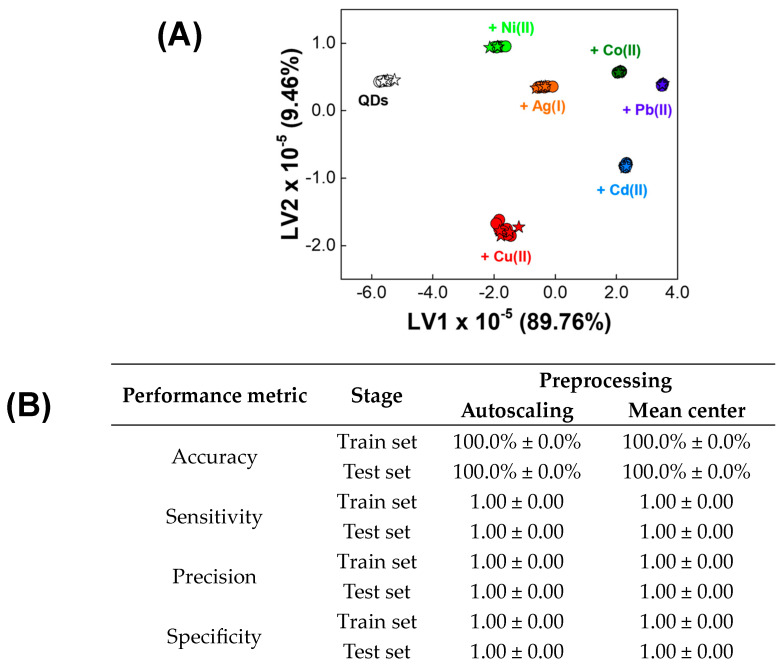
The results of PLS-DA classification of metal ions based on EEM data of QDs. (**A**) Exemplary PLS-DA score plot showing discrimination obtained for train and test sets (circles and stars symbols, respectively). (**B**) The performance of PLS-DA models (mean ± SD, *n* = 3).

An exemplary PLS-DA score plot showing discrimination obtained for both the train and test sets is presented in Figure 3A (and corresponding dendrogram in Figure S2 in ESI). Regardless of the applied preprocessing method, the minimum value of classification error of cross-validation was reached for PLS-DA models with three LVs. Interestingly, the excellent performance of PLS-DA models in a task of metal ion classification was achieved for both autoscaled and mean-centered EEM data (Figure 3B). Thus, all samples in the train set were correctly classified, as evidenced by an accuracy of 100%. Consequently, sensitivity, precision and specificity of PLS-DA models established using both data pretreatment methods for train sets were of 1.00. Great generalization abilities of both PLS-DA models were also confirmed using an independent test set, as the accuracy of 100% and sensitivity, precision, and specificity of 1.00 were achieved in this case. The results obtained did not differ depending on the randomly selected train set and test set, which indicates the reliability of the PLS-DA models obtained using both methods of data preprocessing. However, it is worth noting that the obtained loadings are much more difficult to interpret when autoscaling as the pre-processing step was applied (Figure S1 in ESI). Therefore, according to well-known guidelines for spectral data processing, the recommended data pretreatment method is mean-centering. If there are chemical and experimental reasons to use autoscaling, it is preferable to compare the quality of the PLS-DA models obtained using both methods and to select the simpler and better interpretable model for the final application.

### 3.2. Quantitative Analysis of Metal Ions

The previous section dedicated to qualitative analysis not only allowed us to perform the recognition of six metal ions but also showed a diverse, more or less complicated, impact on the EEM fluorescence response of QDs towards these analytes. Therefore, for quantitative analysis, we divided metal ions into subgroups according to the observed effects, i.e., only fluorescence quenching, fluorescence quenching with red-shift, and fluorescence quenching with baseline changes (Figure 2). In addition, preliminary studies estimating the range of the linear response were performed for each group (see Figure S3 in the ESI).

To perform the quantitative analysis, excitation–emission matrices of QDs acquired in the presence of metal ions at various concentration levels (see ‘Quantitative analysis experiments’ section) were processed with unfolded Partial Least Squares (PLS) regression. The PLS algorithm is identical to PLS-DA, however, in this case, LVs correlate descriptive variables with quantitative information (e.g., analyte concentration) [32,33,34]. Since EEM fluorescence data are of second-order (an individual sample can be described by a data matrix), we decided to compare the performance of this classical approach with n-way PLS (N-PLS), which is an extension of PLS regression to multiway data [28]. The number of LVs in each model was established based on the Root Mean Square Error of Cross Validation (RMSECV). The developed models were characterized by the linear fitting of the predicted values of metal ion concentration (y_PRED_) to the reference values (y_REAL_). Therefore, the quality of PLS and N-PLS models was evaluated based on respective regression parameters (‘a’, ‘b’, ‘R^2^’) calculated for both the train and test sets, assuming that in an ideal case slope (‘a’) is equal to 1, intercept (‘b’) is 0, and determination coefficient (‘R^2^’) is 1. To better assess the performance of PLS and N-PLS models in metal ions determination, the Root Mean Square Error of Calibration (calculated for train set samples, RMSEC) and Root Mean Square Error of Prediction (calculated for test samples, RMSEP) were also calculated. The definition of Root Mean Square Error (RMSE) is presented in Table S2 (see the ESI).

#### 3.2.1. Quantitative Analysis for Metal Ions Only Quenching QDs Fluorescence

**Figure 4:**
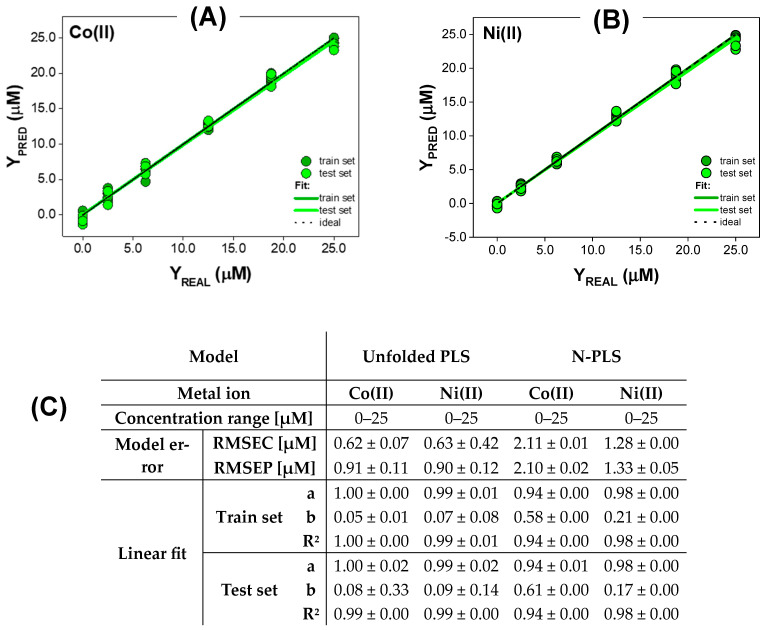
The results of PLS determination of Co(II) and Ni(II) ions influencing EEMs of QDs. (**A**,**B**) Exemplary comparison graphs of predicted vs. real concentration of Co(II) and Ni(II), respectively. Results presented for both train and test sets; the dashed line corresponds to the ideal comparison line (y = x, i.e., a = 1, b = 0, R^2^ = 1). (**C**) The performance of PLS models calculated based on the comparison graphs for each parameter shown as mean ± SD (n = 3).

The first group of metal ions for which we checked the possibility of quantitative determination and compared the performance of PLS and N-PLS models were those causing only the quenching of the fluorescence of QDs, namely Co(II) and Ni(II). The quality of the obtained PLS and N-PLS models is presented in Figure 4. The concentration range in which PLS and N-PLS models operate, both for Co(II) and Ni(II), is 0–25 µM. For Co(II) ions, the slope and determination coefficient reached ideal values for both the train and test sets when the EEM data were modeled using unfolded PLS (‘a’ and ‘R^2^’ of 1.00 for train set, ‘a’ of 1.00 and ‘R^2^’ of 0.99 for test set). Slightly worse models, but also characterized by close to the ideal values of ‘a’ and ‘R^2^’, were obtained in the case of the N-PLS model, and were 0.94 for both the train and test sets. The achieved accuracy differed between the PLS model and N-PLS. In the case of the PLS model, the average ‘b’ value obtained for the train set was 0.05, and for the test set was 0.08, but the standard deviation indicates lower reliability at the prediction stage. In turn, the intercept is higher in the case of the N-PLS model for Co(II) ions determination (0.58 for the train set, 0.61 for the test set), showing its worse precision, but the standard deviation indicates greater reliability of the results obtained using this method. The average RMSEC and RMSEP values are lower in the case of the PLS model (0.62 µM and 0.91 µM for the train and test set, respectively). In contrast, when N-PLS was applied to the EEM data of QDs influenced by Co(II) ions, RMSEC and RMSEP values of 2.11 µM and 2.10 µM were obtained. The difference between the obtained RMSEC and RMSEP values indicates a higher tendency to overfitting of the PLS model, compared to the N-PLS one. This is also reflected in the fact that PLS results slightly depended on the randomly selected train and test sets, as evidenced by the standard deviation. The reason for this might be the fact that samples are described by many more variables after the unfolding of EEMs, and a larger train set should be used to achieve better generalization abilities of the PLS model. Nevertheless, it should be emphasized that the overall performance of the PLS model for the quantification of Co(II) ions based on their influence on QDs’ excitation–emission matrix is superior to that of the N-PLS model. Similar trends can also be observed in the case of Ni(II) ions. Even though the linear fit parameters (‘a’, ‘b’, ‘R^2^’) for the train and test sets are near-ideal in the case of the PLS model (‘a’ and ‘R^2^’ of 0.99 for the train and test sets, ‘b’ of 0.07 for the train set, and 0.09 for test set), the repeatability of the created models was slightly worse, compared to N-PLS (Figure 4C). Once again, the difference between RMSEC and RMSEP, and their standard deviations, were higher in the PLS model (0.63 µM and 0.90 µM, respectively), which shows a higher tendency to overfitting compared to the N-PLS model (RMSEC was 1.28 µM and RMSEP was 1.33 µM). However, the overall performance of the PLS model for the quantification of Ni(II) ions was better than N-PLS. Therefore, the application of unfolded PLS is recommended for the processing of the EEMs of QDs quenched by metal ions. The presented approach can be used for the quantification of Co(II) and Ni(II) ions at the micromolar level.

#### 3.2.2. Quantitative Analysis for Metal Ions Quenching QDs Fluorescence with Red-Shift

**Figure 5:**
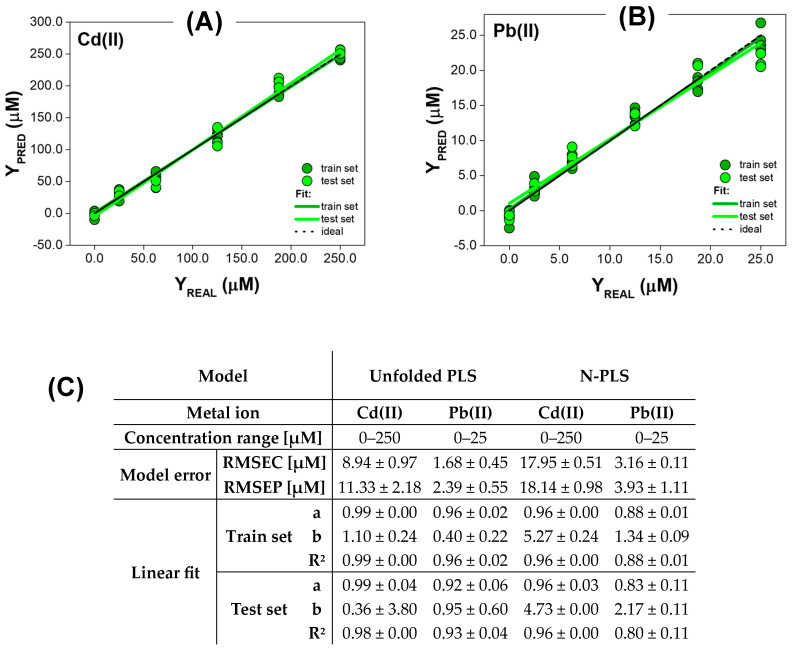
The results of PLS determination of Cd(II) and Pb(II) ions influencing EEMs of QDs. (**A**,**B**) Exemplary comparison graphs of predicted vs. real concentration of Cd(II) and Pb(II), respectively. Results presented for both train and test set; the dashed line corresponds to the ideal comparison line (y = x, i.e., a = 1, b = 0, R^2^ = 1). (**C**) The performance of PLS models calculated based on the comparison graphs, for each parameter shown as mean ± SD (*n* = 3).

The performance of PLS and N-PLS applied to EEM data of QDs influenced by Cd(II) and Pb(II) ions is presented in Figure 5. Both of these ions, apart from quenching the fluorescence of the nanocrystal, also caused a shift in the emission maximum towards longer wavelengths. First of all, both PLS and N-PLS models established for Cd(II) ions determination operate in a very wide concentration range of 0–250 µM. The linear fir parameters ‘a’ and ‘R^2^’ obtained with PLS were near-ideal for both the train set and test set (‘a’ was 0.99 for the train set and test set, while ‘R^2^’ was 0.99 for the train set and 0.98 for test set). However, the precision achieved is much worse (‘b’ of 1.10 for the train set and 0.36 for test set), especially when the very high standard deviation of ’b’ parameter for the test set is considered (Figure 5C). In addition, RMSEC of 8.94 µM and RMSEP of 11.33 µM show that the determination of Cd(II) ions with the presented approach is unsatisfactory. The performance of the N-PLS model in this task is even worse. Thus, the slope and determination coefficient was 0.96 for both the train and test set, while the intercept of 5.27 was obtained in the case of the train set and 4.73 for the test set. The achieved RMSEC and RMSEP for the N-PLS model are much higher than in the case of unfolded PLS, thus, 17.95 µM and 18.14 µM, respectively. This proves that the use of the influence of Cd(II) ions on the EEM spectrum of quantum dots does not allow for a precise quantitative analysis of this analyte in the tested concentration range, regardless of the data processing method used.

In the case of Pb(II) ions, PLS and N-PLS models operating in the concentration range of 0–25 µM were established (Figure 5B,C). Similarly to metal ions, which only quenched the fluorescence of QDs, the performance of the N-PLS model was slightly worse than the PLS one. Thus, for the train set, the slope and determination coefficient were 0.88, while the intercept was 1.34. The worsening of linear fit parameters in the case of the test set was observed when EEM data were modelled by N-PLS (‘a’ was 0.83, ‘b’ was 2.17, and ‘R^2^’ was 0.80). Moreover, the N-PLS model was characterized by RMSEC of 3.16 µM and RMSEP of 3.93 µM. In contrast, the use of unfolded PLS allowed the reduction of RMSEC and RMSEP values to 1.68 µM and 2.39 µM, respectively, providing higher precision of the determination. The difference in these values shows that expanding the train set could be beneficial for improving the generalization ability of the PLS model. The accuracy of Pb(II) ions’ concentration prediction was satisfactory for the train set (‘b’ was 0.40), but worse results were obtained using the independent test set (‘b’ was 0.95). Nevertheless, the obtained ‘a’ and ‘R^2^’ in case of PLS modeling of 0.96 for the train set, and around 0.92 for the test set, indicate good accuracy and precision of the developed models. In conclusion, the classical PLS model is better at processing fluorescence data from QDs when the observed effect in their EEM spectrum in the presence of metal ions is fluorescence quenching with red-shift.

#### 3.2.3. Quantitative Analysis for Metal Ions Quenching QDs Fluorescence with Baseline Changes

**Figure 6:**
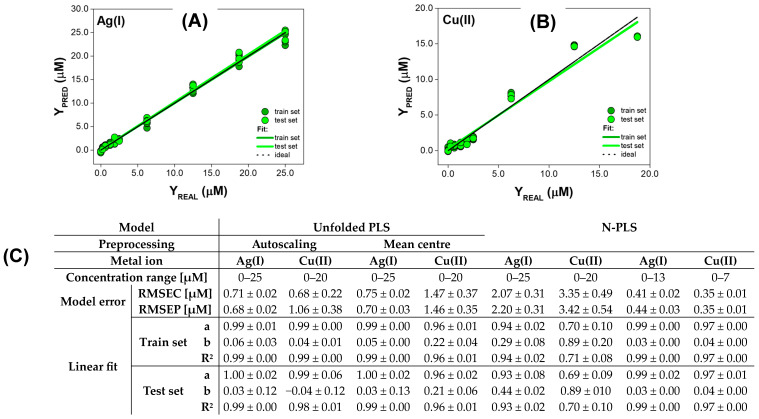
The results of PLS determination of Ag(I) and Cu(II) ions influencing EEMs of QDs. (**A**,**B**) Exemplary comparison graphs of predicted vs. real concentration of Ag(I) and Cu(II), respectively. Results presented for both train and test sets; the dashed line corresponds to the ideal comparison line (y = x, i.e., a = 1, b = 0, R^2^ = 1). (**C**) The performance of PLS models calculated based on the comparison graphs for each parameter shown as mean ± SD (n = 3).

The last group of metal ions for which the performance of unfolded PLS and N-PLS was compared were Ag(I) and Cu(II) ions that quenched the fluorescence of QDs and induced the changes in the baseline (Figure 6). Since part of the useful information for the quantitative determination of Ag(I) and Cu(II) may be contained in baseline changes, we decided to additionally compare the performance of the PLS method with autoscaled and mean-centred EEM fluorescence data.

The concentration range of Ag(I) ions, in which models operated, differed for unfolded PLS and n-way PLS. Thus, in the case of the PLS model, it was possible to determine analytes in the range of 0–25 µM with both autoscaled and mean-centred data, while a narrower concentration range of 0–13 µM was obtained with N-PLS. This might be related to the fact that, at higher concentrations of Ag(I) ions, the baseline changes are more significant for analyte determination and the PLS method is better at describing them. Nevertheless, the N-PLS model was characterized by near-ideal linear fit parameters for both the train set and test set, which proves its great generalization capabilities (‘a’ and ‘R^2^’ of 0.99, and ‘b’ of 0.03 for both train and test set). In addition, the model error for the train set and test set were low, and their values were comparable, which indicates that the N-PLS method is not prone to overfitting (RMSEC was 0.41 µM, RMSEP was 0.44 µM) and can be successfully applied to determine Ag(I) ions. In the case of the PLS model, great precision was achieved regardless of the data pretreatment step, as evidenced by the slope and determination coefficient of 0.99 for the train set, and ‘a’ of 1.00 and ‘R2’ of 0.99 for the test set. However, the accuracy of PLS models obtained with both autoscaled and mean-centred data for test samples was slightly worse than for the train set, given the standard deviation of ‘b’ parameter (Figure 6C). RMSEC and RMSEP values obtained with autoscaled and mean-centred data are low and similar, showing that both data pretreatment methods allowed us to obtain the PLS model with a greater ability to determine Ag(I) ions in the broader concentration range than in the case of N-PLS. Moreover, the comparability of the obtained values proves that the PLS model was not prone to overfitting, despite the large number of descriptive variables resulting from the unfolding of EEMs (RMSEC was 0.71 µM and 0.75 µM, and RMSEP was 0.68 µM and 0.70 µM for autoscaled and mean-centred data, respectively).

In the case of Cu(II) ions, the N-PLS model operating in the concentration range of 0–7 µM was obtained, however, its great performance must be noted. The accuracy and precision obtained for the train set were convergent with those achieved for test samples (‘a’ and ‘R^2^’ were 0.97 for both the train and test sets, while ‘b’ was 0.04), showing that an appropriate relationship between the information contained in EEMs and the concentration of Cu(II) was established during the model calibration, and great generalization abilities were also achieved for concentration prediction. The RMSE of Cu(II) concentration determination for the train set and test set was very low, indicating the usefulness of the N-PLS method for the quantification of the analytes (RMSE and RMSEP of 0.35 µM). In contrast, when EEM data of QDs acquired in the presence of Cu(II) ions were processed with PLS, the broader concentration range in which the model operated was achieved, i.e., 0–20 µM, for both autoscaling and mean-centering. These results are convergent with previous observations for Ag(I) ions. However, in this case, the performance of the PLS method differed depending on the data pretreatment method applied. When the data were mean-centred, higher RMSEC and RMSEP values of 1.47 µM and 1.46 µM were achieved, respectively. Moreover, even though the precision was high for both the train set and test set (‘R^2^’ was 0.96 for mean-centering, and 0.99 for autoscaling), the PLS model obtained with autoscaled data exhibited problems related to accuracy (‘b’ around 0.22 for the train set and test set). In addition, the lowering in RMSEC and RMSEP values was observed when the EEM fluorescence data of the QDs-Cu(II) system were autoscaled (0.68 µM and 1.06 µM, respectively). However, it should be noted that the difference between these values is greater than in the case of mean-centering, and the repeatability of models obtained using a randomly selected train set and test set is worse. In accordance with that, using more train samples might be beneficial for achieving better performance of Cu(II) ions determination with unfolded PLS when autoscaling is applied. For wide concentration ranges (0–25 μM for Ag(I) and 0–20 μM for Cu(II) ions), again, processing of unfolded data with PLS is favorable, as it performs much better in the case of both metal ions’ quenching QDs fluorescence with baseline changes, especially in the case of the Cu(II) increase in the error several times (from ca 0.7 μM to ca 3.4 μM), and a significant drop in the determination coefficient (‘R^2^‘ at the level of 0.7) is noticeable and shows a significant distinction between models’ performance. All these findings show that the optimization of PLS models is a crucial step in the development of the QDs sensing system, providing the possibility to gain better precision, higher accuracy, and a wider concentration range.

## 4. Conclusions

Multi-responsive probes are attractive recognition molecules/structures due to their ability to sense more than one analyte. While the synthesis of hosts with two or more recognition moieties can be complicated, laborious, and sometimes even impossible, an alternative approach based on non-specific receptors interacting with the target analytes and providing a multiplexed response can provide successful multiparameter detection and/or quantification. We showed previously [17] how multiparameter sensing can be realized with the use of only a single probe type. Such a virtual receptor array produced characteristic fluorescent response patterns that were processed by PLS-DA for the satisfactory identification of all investigated neurotransmitters, significantly influenced the sensing performance, and anticipated that the optimization of preprocessing type and data structure may be beneficial. Therefore in the current work, we investigated various parameters of the PLS method used for the multispectral fluorescence detection of heavy metals by QDs. The presented sensing strategy is based on the fact that selected metal ions exhibit different quenching mechanisms of the QDs’ fluorescence, manifested by their diverse influence on the fluorescence spectrum of this nanomaterial (i.e., the degree of the fluorescence quenching and spectral shifts). By utilizing the multispectral fluorescence as a detection technique, we captured the information on the alterations of the fluorescence signal of QDs caused by metal ions and showed how chemometric modeling of obtained excitation–emission matrices can be used for the identification of selected analytes and their quantification.

In the discrimination of the studied set of heavy metals, the type of preprocessing (autoscaling vs. mean-centering) did not influence the identification results in terms of all applied performance metrics: accuracy, sensitivity, precision, and specificity. Various mechanisms involved in the interaction of metal ions and quantum dots entailed distinct effects visible on the EEM spectrum: fluorescence quenching with or without red-shift, and with or without baseline changes. This observation shows that even studying 0th or 1st-order QDs’ fluorescent response, it is often possible to obtain a better result by simply taking into account the type of calibration signal. It also proves that multiplexing is possible and beneficial when multispectral fluorescence data are collected. However, it can be surprising, that, in the case of all heavy metal ions studied in this work, N-PLS operating on higher order data structure, thus being more informative, provided worse performance than unfolded PLS. We stress that this effect can be limited to quite simple sensing systems where single analytes are present in the tested samples–multiple sensing of many analytes in mixtures, which can demand the consideration of higher data structures, which will be investigated in our future work.

## Data Availability

The raw data supporting the conclusions of this article will be made available by the authors on request.

## Supplementary Materials

The following supporting information can be downloaded at: https://www.mdpi.com/article/10.3390/ma17194766/s1, Scheme S1: Schematic representation of EEM data processing by PLS-DA in the case of qualitative analysis, Table S1: Definitions of quality performance metrics (accuracy, sensitivity, precision, specificity) used to assess the quality of PLS-DA models, Figure S1: Loading plots for PLS-DA models obtained with (A–C) autoscaled, (D–F) mean centered data, Figure S2: Hierarchical cluster analysis (HCA) showing discrimination of QDs samples containing investigated metal ions. Ward’s method and Mahalanobis distance for mean-centered data were applied, Figure S3: Preliminary results showing the change in QD fluorescence intensity as a function of the logarithm of metal ion concentration, Scheme S2: Schematic representation of EEM data processing by PLS in the case of quantitative analysis, Table S2.: The definition of root mean square error (RMSE): yPRED is the predicted value for the n-th sample, yREAL is the true value known before, and N is total number of samples.
