# Supplementary material for: The Performance of Partial Least Squares Methods in Virtual Nanosensor Array—Multiple Metal Ions Sensing Based on Multispectral Fluorescence of Quantum Dots
Source: Materials (Basel). 2024 Sep 28;17(19):4766. doi: 10.3390/ma17194766 (PMC11477732; doi:10.3390/ma17194766)
Supplement: Supplementary file 1 [file materials-17-04766-s001.zip › materials-3152006-supplementary.pdf]

Electronic Supplementary Information for:

The Performance of Partial Least Squares Methods in Virtual Nanosensor Array—Multiple Metal Ions Sensing Based on Multispectral Fluorescence of Quantum Dots

*Klaudia Głowacz, Mikołaj Cie´slak and Patrycja Ciosek-Skibi ´nska **

Chair of Medical Biotechnology, Faculty of Chemistry, Warsaw University of Technology,

Noakowskiego 3,

00-664 Warsaw, Poland; klaudia.glowacz@pw.edu.pl (K.G.)

* Correspondence: patrycja.ciosek@pw.edu.pl

1. Qualitative analysis of metal ions

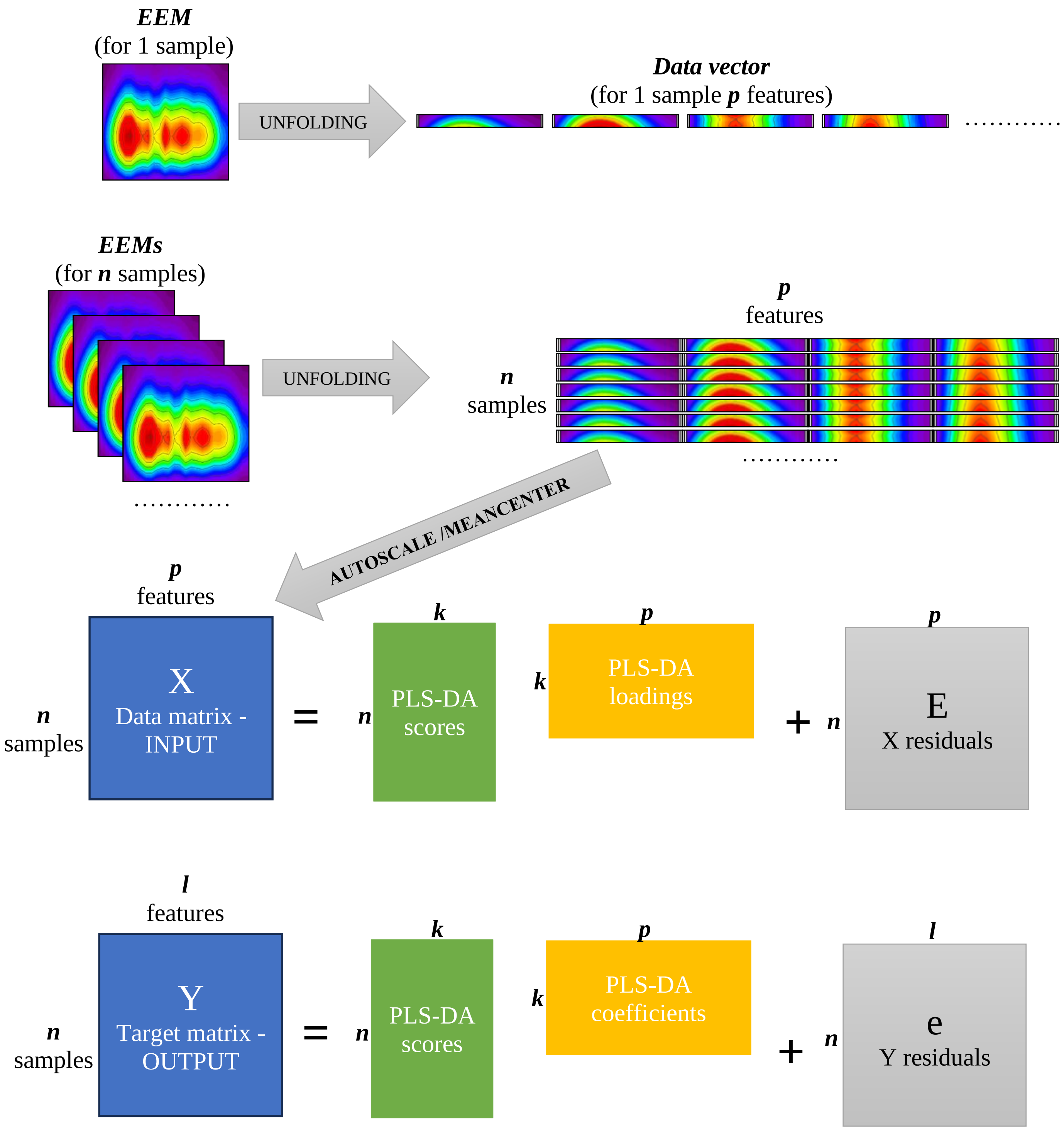

Scheme S1. Schematic representation of EEM data processing by PLS-DA in the case of qualitative analysis.

Table S1. Definitions of quality performance metrics (accuracy, sensitivity, precision, specificity) used to assess the quality of PLS-DA models. The values of TN, FN, FP and TP for each investigated class (type of sample) were obtained from the PLS-DA model response in the form of a confusion matrix, for both train and test set (simplified confusion matrix for binary classification given below). TP – true positives (number of samples of a given class correctly predicted as this class), TN – true negatives (the number of samples correctly not assigned to a given class), FN – false negatives (number of samples of a given class incorrectly predicted as other class), FP – false positives (number of samples of other class incorrectly predicted as a given class).

		**The class predicted by PLS-DA model**	
		**A**	**B**
**An actual class**	**A**	*TP*	*FN*
	**B**	*FP*	*TN*

$$accuracy = \frac{(TP + TN)}{(TP + TN + FP + FN)}$$

$$sensitivity = \frac{TP}{(TP + FN)}$$

$$precision = \frac{TP}{(TP + FP)}$$

$$specificity = \frac{TN}{(TN + FP)}$$

Figure S1. Loading plots for PLS-DA models obtained with (A-C) autoscaled, (D-F) mean centered data. (A, D) LV1. (B, E) LV2. (C, F) LV3.

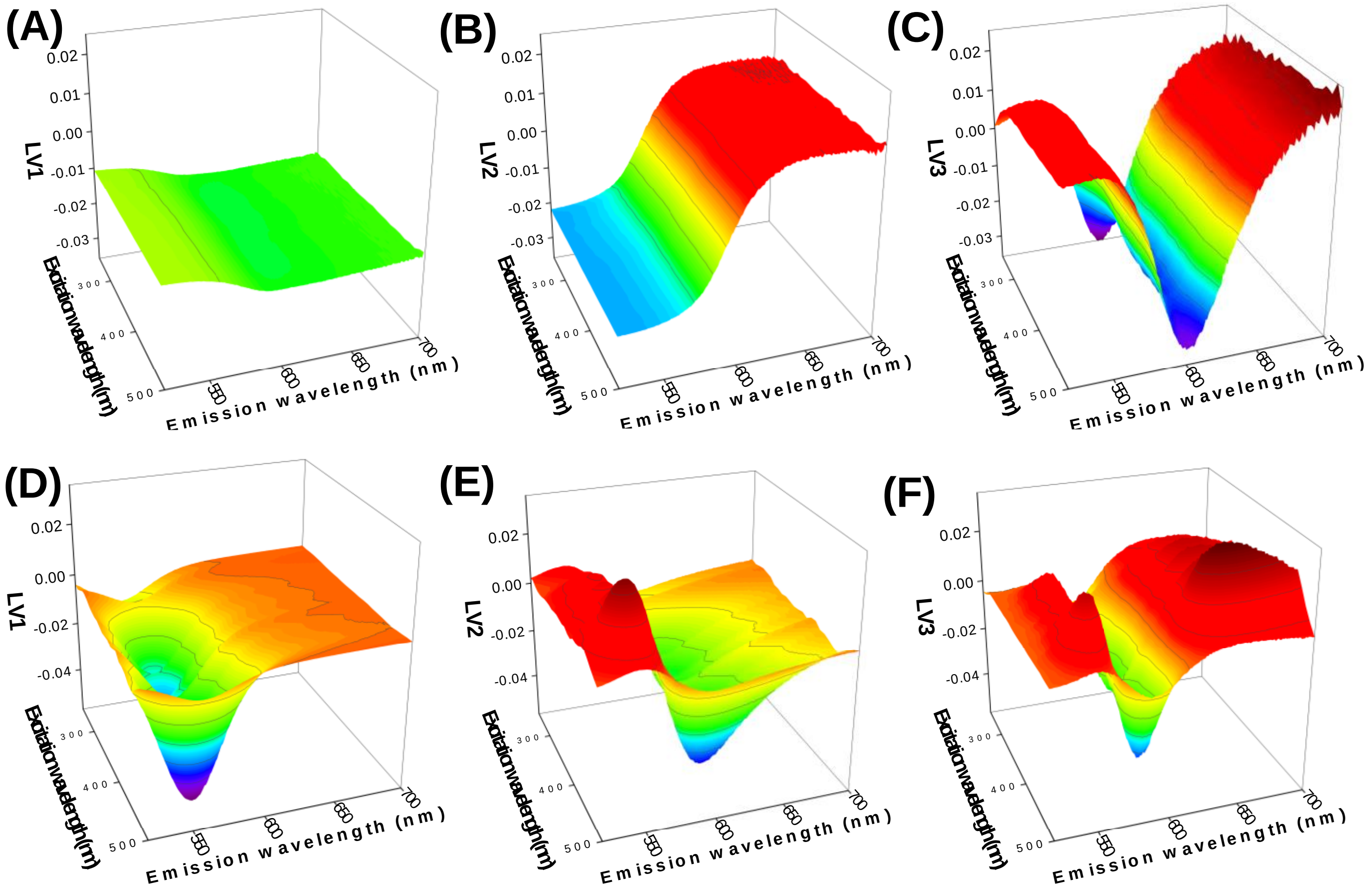

Figure S2. Hierarchical cluster analysis (HCA) showing discrimination of QDs samples containing investigated metal ions. Ward's method and Mahalanobis distance for mean-centered data were applied.

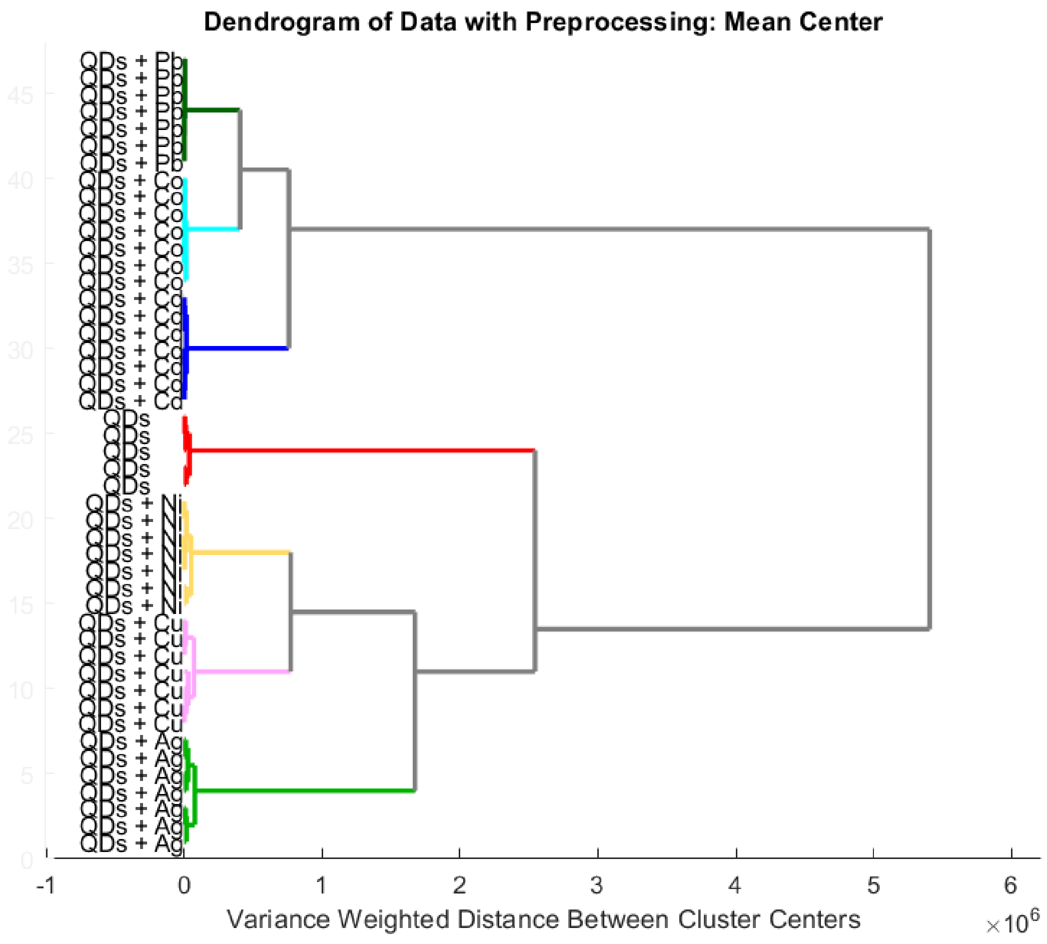

2. Quantitative analysis of metal ions

Figure S3. Preliminary results showing the change in QD fluorescence intensity as a function of the logarithm of metal ion concentration: (A) Co(II), (B) Ni(II), (C) Cd(II), (D) Pb(II), (E) Ag(I) and (F) Cu(II). The graphs were prepared based on fluorescence spectra of QDs acquired in the presence of metal ions at levels from 10^{-1} M to 10^{-9} M (λ_{ex} = 290 nm, λ_{em}: 310-700 nm, fluorescence intensity presented as mean ± SD; n = 3).

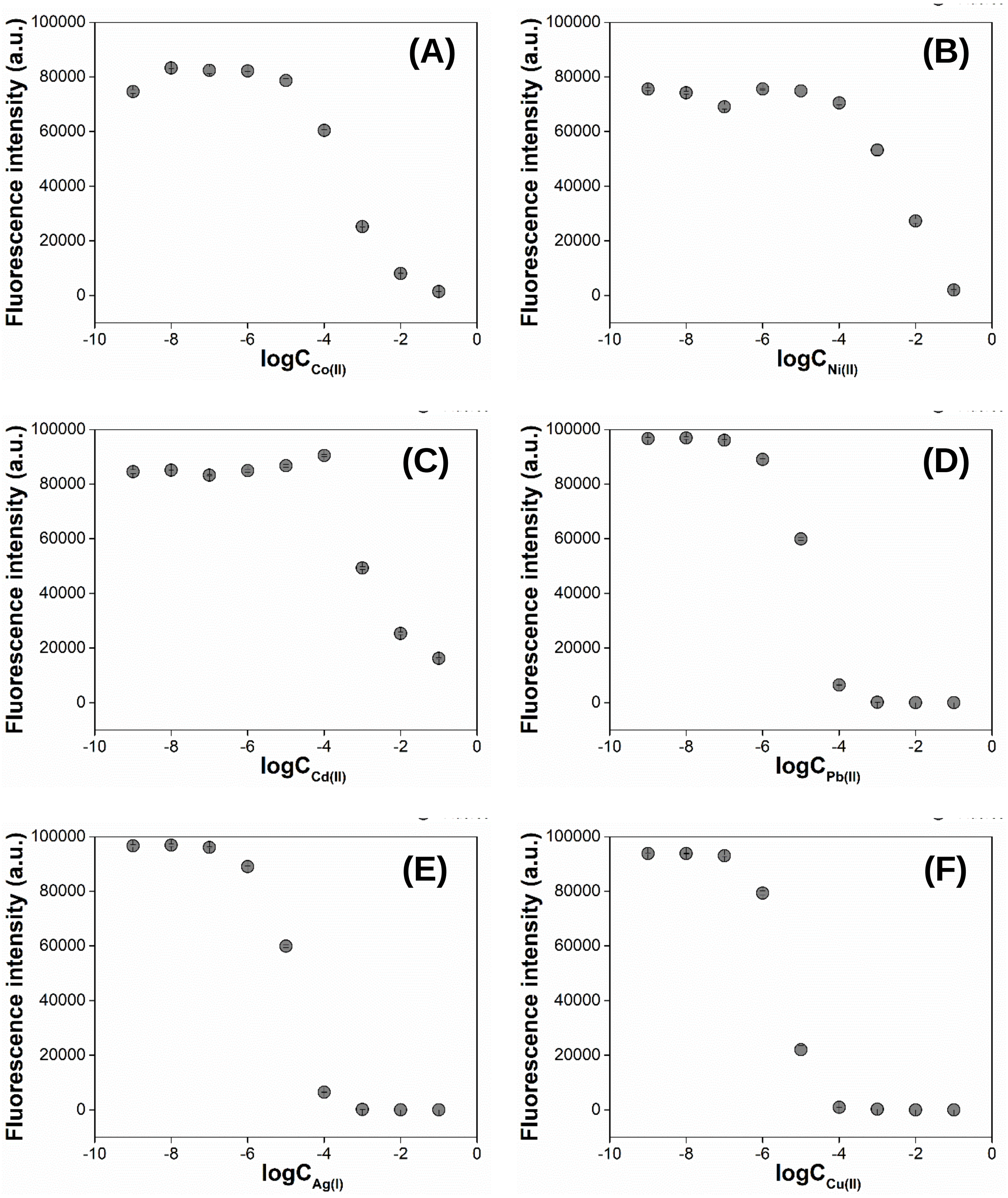

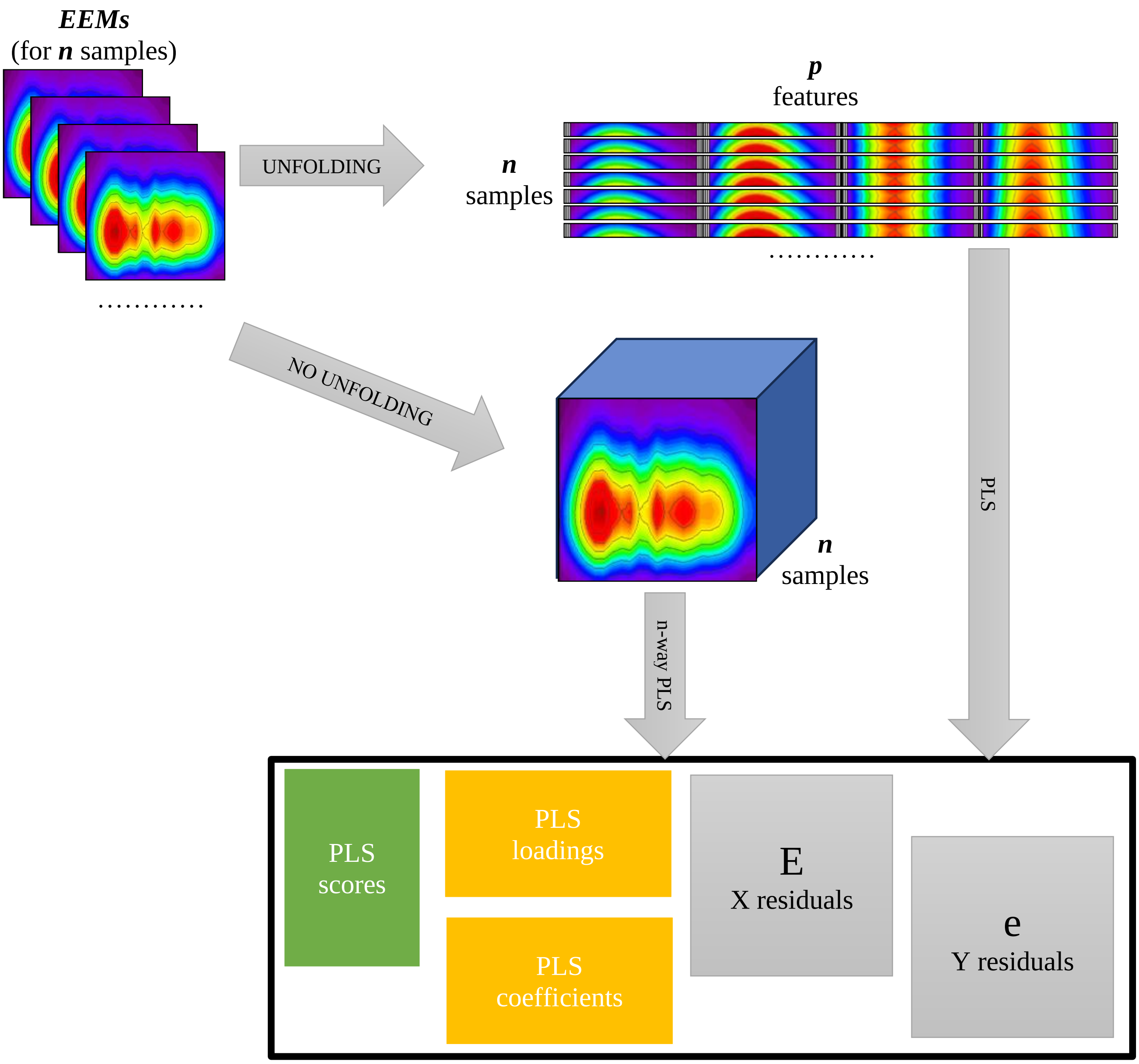

Scheme S2. Schematic representation of EEM data processing by PLS in the case of quantitative analysis.

Table S2. The definition of root mean square error (RMSE): y_{PRED} is the predicted value for the *n*-th sample, y_{REAL} is the true value known before, and *N* is total number of samples. RMSEC, RMSECV, and RMSEP are calculated for calibration (train) set, cross-validation data, and test (validataion) set, respectively.

$$RMSE = \sqrt{\frac{1}{N-1}\sum_{n=1}^{N}(y_{REAL} - y_{PRED})^2}$$
